# A reliable numerical investigation of an SEIR model of measles disease dynamics with fuzzy criteria

**DOI:** 10.1038/s41598-023-42953-x

**Published:** 2023-09-22

**Authors:** Fazal Dayan, Nauman Ahmed, Muhammad Rafiq, Ali Raza, Ilyas Khan, Elsayed Mohamed Tag eldin

**Affiliations:** 1 Department of Mathematics, School of Science, University of Management and Technology, Lahore, Pakistan; 2 Department of Mathematics and Statistics, The University of Lahore, Lahore, Pakistan; 3 Department of Computer Science and Mathematics, Lebanese American University, Beirut, Lebanon; 4 Department of Mathematics, Faculty of Science & Technology, University of Central Punjab, Lahore, Pakistan; 5 Department of Mathematics, Govt. Maulana Zafar Ali Khan Graduate College Wazirabad, Punjab Higher Education Department (PHED), Lahore, 54000 Pakistan; 6 Department of Mathematics, College of Science Al-Zulfi, Majmaah University, Al-Majmaah, 11952 Saudi Arabia; 7 Faculty of Engineering and Technology, Future University in Egypt, New Cairo, 11835 Egypt

**Keywords:** Biological techniques, Biotechnology, Computational biology and bioinformatics, Diseases

## Abstract

The terms susceptibility, exposure, infectiousness, and recovered all have some inherent ambiguity because different population members have different susceptibility levels, exposure levels, infectiousness levels, and recovery patterns. This uncertainty becomes more pronounced when examining population subgroups characterized by distinct behaviors, cultural norms, and varying degrees of resilience across different age brackets, thereby introducing the possibility of fluctuations. There is a need for more accurate models that take into account the various levels of susceptibility, exposure, infectiousness, and recovery of the individuals. A fuzzy SEIR model of the dynamics of the measles disease is discussed in this article. The rates of disease transmission and recovery are treated as fuzzy sets. Three distinct numerical approaches, the forward Euler, fourth-order Runge-Kutta, and nonstandard finite difference (NSFD) are employed for the resolution of this fuzzy SEIR model. Next, the outcomes of the three methods are examined. The results of the simulation demonstrate that the NSFD method adeptly portrays convergent solutions across various time step sizes. Conversely, the conventional Euler and RK-4 methods only exhibit positivity and convergence solutions when handling smaller step sizes. Even when considering larger step sizes, the NSFD method maintains its consistency, showcasing its efficacy. This demonstrates the NSFD technique’s superior reliability when compared to the other two methods, while maintaining all essential aspects of a continuous dynamical system. Additionally, the results from numerical and simulation studies offer solid proof that the suggested NSFD technique is a reliable and effective tool for controlling these kinds of dynamical systems.The convergence and consistency analysis of the NSFD method are also studied.

## Introduction

Mathematical modeling of infectious diseases entails the use of mathematical equations and tools to understand the spread and dynamics of diseases within populations. It seeks to comprehend transmission patterns, forecast the future course of an epidemic, assess the effectiveness of control measures, and inform public health actions. To simulate and assess the course of infectious diseases, mathematical models can combine many aspects such as population demographics, disease parameters, transmission channels, and intervention tactics. These models can assist policymakers and researchers in making educated decisions about disease prevention, control, and mitigation techniques. In epidemiology, mathematical modeling is used to explain how diseases spread and what impact they have on populations. This covers a wide range of academic fields, including sociology, philosophy, engineering, biology, and mathematics. Epidemiological models enable a deeper comprehension of the spread of the illness and its prevention measures^1^. A model for predicting the behavior of the disease was developed, and it was one of the first mathematical epidemiological models^2^. Following that, a large number of models were developed, tested, and utilized both qualitatively and quantitatively to research a wide range of infectious diseases. Several conventional schemes, including forward Euler, Runge-Kutta, and similar methods, can encounter difficulties such as the occurrence of oscillations, chaos, and erroneous steady states. To circumvent these numerical instabilities, an alternative approach is to construct schemes utilizing the nonstandard finite-difference method. By adopting this method, it becomes possible to avoid the aforementioned issues and ensure greater stability in numerical computations. This technique, pioneered by Mickens^3^ has resulted in the development of new numerical schemes that preserve physical qualities such as stability, positivity, and boundedness, etc. Verma and Kayenat investigated Mickens’ NSFD theory and demonstrated its convergence by applying these schemes to Lane Emden equations^4^. Verma and Kayenat conducted an investigation on the generalized Burgers–Huxley (GBH) equation, employing the NSFD technique. To demonstrate the superior performance of the proposed approach, the maximum error of the NSFD solutions is compared with that of various alternative methods. Additionally, the computational time required for the computations is compared, and the results showcased that the NSFD scheme yielded accurate results within a short duration of a few seconds, thus offering significant time savings^5^. Mickens and Washington created a new NSFD strategy for a heat transfer hyperbolic partial differential equation (PDE)^6^. Hoang et al. employed Mickens’ methods to develop NSFD schemes for multiple epidemiological models related to the spread of computer viruses and malware. The suggested NSFD schemes were thoroughly examined to assess their properties of positivity, boundedness, and global asymptotic stability^7^. Conte et al. investigated the benefits of employing NSFD numerical techniques for the solution of both ordinary differential equations (ODEs) and partial differential equations (PDEs)^8^. Hoang developed and tested NSFD techniques for a well-known virus-patch dynamic model^9^. Hoang utilized the NSFD technique to examine the qualitative dynamical characteristics of a generalized hepatitis B epidemic model, as well as its corresponding discretized model with dynamic consistency^10^. Fatima et al. created the SLBQRS model and solved it using the NSFD, the forward Euler technique, and the order 4 Runge–Kutta method^11^. To overcome the population dynamics problem posed by the SEIQV outbreak, Ahmed et al. created the NSFD scheme^12^. Many other researchers applied the NSFD scheme in multiple directions^13–20^, just to mention a few.

Despite the presence of a safe and cost-effective vaccine, measles continues to be a significant cause of death, particularly among children, as it is an extremely contagious and potentially fatal illness caused by the morbillivirus. Measles is easily transmitted from one person to another through infected individuals’ coughs and sneezes. Additionally, transmission can occur through direct contact with the mouth or surfaces that have been contaminated^21^. It is a leading cause of death in many nations, particularly among children^22^. After an incubation period lasting approximately 8 to 12 days, measles manifests with symptoms consisting of fever, cough, coryza (nasal inflammation), and conjunctivitis (inflammation of the eye). Subsequently, a characteristic rash emerges, starting on the face and neck and then spreading to other parts of the body^23^. Mathematical models were critical in assisting with measles elimination efforts and providing insights into the transmission of measles within a population. They aid in the identification of crucial transmission parameters such as contact rates, vaccination coverage, and population demographics. Models enable researchers to quantify the impact of these factors on disease dynamics, which helps to inform disease control and prevention measures. Numerous models have been developed and examined using diverse methodologies to enhance our understanding of the transmission dynamics and control of the disease. A measles disease model was utilized to explore the prevalence and control of the disease in Senegal, and the Runge–Kutta fourth order approach was employed to numerically solve the model^24^. Sowole et al. created a mathematical model to examine the dynamics of the measles disease using data from Nigeria. In order to investigate the prevalence and management of measles disease, a control measure was implemented within the susceptible and exposed population groups^25^. In order to study the impact of vaccination on containing the measles epidemic, Liuyong Pang et al. proposed an SEIR model. An NSFD strategy has been created by Ahmed et al. to solve the SEIR measles epidemic model with diffusion^26^. Tilahun et al. created a stochastic model of measles transmission dynamics using a two-dose vaccination strategy. The study involved an investigation into the conditions ensuring the positivity of solutions, the invariant region of the solution, the existence and stability of equilibrium points in the model, and the sensitivity analysis of the model^27^. A network model with periodic transmission rate was developed to analyze the effects of heterogeneity and waning immunity on measles transmission and to theoretically examine the threshold dynamics^28^. Mathematical modeling of infectious disease is studied a lot in recent times^29–35^, for example.

Zadeh established the idea of fuzzy theory in 1965^36^. The utilization of fuzzy theory in mathematical modeling is pivotal as it offers a means to address uncertain or ambiguous information within a mathematical framework. Conventional mathematical models assume the precision and accurate measurement or calculation of all variables. However, in numerous real-world situations, variables may be imprecise or difficult to assess, resulting in model uncertainty. The fuzzy theory has found widespread application across various mathematical modeling domains, allowing for the incorporation of fuzzy logic to handle such uncertainties. Ortega et al.^37^ studied a fuzzy dynamical system based epidemic model. The epidemiological issues associated with infectious diseases were predicted using fuzzy logic. A dog model of rabies with a partial vaccination was discussed. Mondal et al. formulated an epidemic model that considered the influence of treatment control^38^. Verma et al. explored a model of Influenza propagation characterized by an asymptotic transmission rate. The rates of disease transmission and mortality were treated as fuzzy sets. Through the utilization of probability measures and fuzzy expected values, they derived the fuzzy basic reproduction number for various subgroups of infected individuals exhibiting different levels of viral loads. Furthermore, a comparative analysis of the basic reproduction numbers between the traditional and fuzzy models was also conducted^39^. Verma et al. conducted an investigation into the transmission dynamics of Ebola virus disease, considering the inherent variability by introducing fuzziness to all biological parameters. Through the utilization of triangular fuzzy numbers to account for imprecision in these parameters, they thoroughly examined the existence and stability of equilibria within the system. Complementing the theoretical findings, numerical experiments were performed to validate the conclusions^40^. Pal and Mahapatra introduced an innovative perspective on the modeling of prey-predator interactions, termed as an imprecise prey-predator model. In this biological model, the concept of interval numbers to represent the uncertainty associated with the model parameters is adopted^41^. Renu et al. constructed a population model using interval values to represent the interrelationships among phytoplankton, zooplankton, and fish populations. This model incorporated a cyrtoid-type functional response^42^. A fuzzy model was created by Jafelice et al. to depict how the number of people with HIV has changed over time and how AIDS symptoms have changed^43^. Boventura and Gonzaga showcased the application of fuzzy theory in performing edge detection on grayscale images^44^. For worm transmission in computer networks, Mishra et al. introduced a fuzzy SIRS model. The analysis explores low, medium, and high severity scenarios for managing worm outbreaks within the computer network, which the attack can also influence, in order to better understand the worm attack. The generated system of equations was solved and simulated using numerical methods^45^. Fuzzy numbers were used in the method Jessica and Filipe designed, and they asserted that skin lesion slide photos could also be used to apply fuzzy numbers^46^. Verma et al. found that the reproduction number in a crisp system does not directly depend on the virus that is causing the disease^39^. A SIR model with imprecise biological factors was presented by Das and Pal^47^. Lefevre et al. discussed applications of fuzzy epidemiological models related to the prevalence of HIV^48^. Dandapanet et al. recently introduced a model by using fuzzy logic and solved by using Laplace-Adoman analysis method and differential transform method for an arbitrary order^49^. Allehiany et al. investigated a Covid-19 model with fuzziness and numerically solved it using the NSFD scheme^50^. Alhebshi et al. looked into a computer virus model that used fuzzy criteria^51^.

The existing models lack comprehensive integration of fuzzy numerical and mathematical techniques. In light of this, we conducted a study focusing on an SEIR model incorporating fuzzy parameters. By employing fuzzy theory, we address the challenges associated with uncertainty quantification in mathematical disease modeling. The inclusion of fuzzy parameters enables a more precise explanation of the transmission of the disease. We formulated numerical solutions for the investigated model using the forward Euler, RK-4, and NSFD methods. The remainder of this research is organized as follows: In Section “Fuzzy SEIR model”, an SEIR model with fuzzy parameters, basic reproduction number (BRN) $R_0$ and fuzzy BRN $R_0^f$ are discussed. Three numerical methods for the model under study are developed in Section “Numerical modeling of fuzzy SEIR model”. In this section, we also talk about the convergence of the NSFD approach. In Section “Numerical simulations”, the numerical simulations of the developed methodologies are presented. The study is wrapped up in Section “Conclusion”.

## Fuzzy SEIR model

Consider the following system describing the SEIR model of measles^26^.

1 $$\begin{aligned} {\frac{dS}{dt}}= & {} {\mu N-\beta SI-\mu S}, \end{aligned}$$

2 $$\begin{aligned} {\frac{dE}{dt}}= & {} {\beta SI-(\mu +\alpha ) E}, \end{aligned}$$

3 $$\begin{aligned} {\frac{dI}{dt}}= & {} {\alpha E-(\mu +\eta ) I}, \end{aligned}$$

4 $$\begin{aligned} {\frac{dR}{dt}}= & {} {\eta I-\mu R}. \end{aligned}$$

The corresponding fuzzy SEIR model to the above model is

5 $$\begin{aligned} {\frac{dS}{dt}}= & {} {\mu N-\beta (\upsilon ) SI-\mu S}, \end{aligned}$$

6 $$\begin{aligned} {\frac{dE}{dt}}= & {} {\beta (\upsilon ) SI-(\mu +\alpha ) E}, \end{aligned}$$

7 $$\begin{aligned} {\frac{dI}{dt}}= & {} {\alpha E-(\mu +\eta (\upsilon )) I}, \end{aligned}$$

8 $$\begin{aligned} {\frac{dR}{dt}}= & {} {\eta (\upsilon ) I-\mu R}. \end{aligned}$$

The above system can be written as

9 $$\begin{aligned} {\frac{dS}{dt}}= & {} {\mu N-\beta (\upsilon ) SI-\mu S}, \end{aligned}$$

10 $$\begin{aligned} {\frac{dE}{dt}}= & {} {\beta (\upsilon ) SI-(\mu +\alpha ) E}, \end{aligned}$$

11 $$\begin{aligned} {\frac{dI}{dt}}= & {} {\alpha E-(\mu +\eta (\upsilon )) I}. \end{aligned}$$

**Figure 1:**
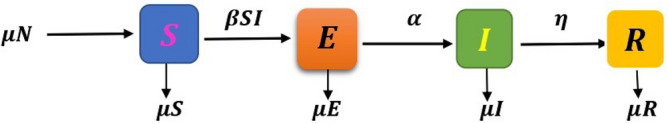
Flowchart of the model.

The flowchart of the model is depicted in Fig. 1.

The parameter $\mu N$, $\beta$, $\alpha$ and $\eta$ are positive constants. *S*, *E*, *I* and *R* are the proportions of susceptible, exposed, infected and recovered individuals respectively. Here $\alpha$ is the rate at which exposed individuals become infected and $\eta$ is the rate of recovery from the infection. $\mu N$ is the influx of individuals into the susceptible, $\mu$ is the natural death rate and $\upsilon$ is the virus load. The fuzzy number $\beta$, is the rate at which susceptible individuals are infected by other infectious individuals and can be defined as

12 $$\begin{aligned} \beta (\upsilon )=\left\{ \begin{array}{ll} 0, &{} \upsilon<\upsilon _m \\ \frac{\upsilon -\upsilon _m}{\upsilon _0-\upsilon _m}, &{} \upsilon _m\le \upsilon \le \upsilon _0 \\ 1, &{} \upsilon _0<\upsilon <\upsilon _M \end{array} \right. \end{aligned}$$

The definition given above demonstrates that the minimum virus load, $\upsilon _m$ necessary for disease transmission will be negligible when the virus load is low. At some point $\upsilon _0$, where it is equal to unity, the transmission rate reaches its maximum. It’s also assumed that the recovery rate $\eta =\eta (\upsilon )$ is a fuzzy number. It will take longer to recover from infection if there is a higher viral load. The following is its fuzzy membership function:

13 $$\begin{aligned} \eta (\upsilon )=\frac{(\eta _0-1)}{\upsilon _M}\upsilon +1, \hspace{1cm} 0\le \upsilon \le \upsilon _M \end{aligned}$$

where the lowest recovery rate is $\eta _0>0$.

### Fuzzy BRN $R_0^f$

By employing the approach of the next-generation matrix, we determine the value of $R_0$. Let $X=[S, E, I]^t$, then $\frac{dX}{dt}=f(x)-v(x)$ where

$$\begin{aligned} f(x)= \left[ \begin{array}{cc} - \beta (\upsilon )SI \\ \beta (\upsilon )SI \\ 0\\ \end{array} \right] \end{aligned}$$

and

$$\begin{aligned} v(x)= \left[ \begin{array}{ccc} \mu S-\mu N \\ (\mu +\alpha ) E \\ (\mu +\eta (\upsilon )) I-\alpha E\\ \end{array} \right] . \end{aligned}$$

Now, *F* and *V* represent the Jacobeans of *f*(*x*) and *v*(*x*) correspondingly, and their expressions are provided as follows.

$$\begin{aligned} F= \left[ \begin{array}{lll} - \beta (\upsilon )I &{} 0 &{} -\beta (\upsilon )S\\ \beta (\upsilon )I &{} 0 &{} \beta (\upsilon )S\\ 0&{}0&{}0\\ \end{array} \right] \end{aligned}$$

and

$$\begin{aligned} V= \left[ \begin{array}{ccc} \mu &{} 0 &{}0\\ 0&{} \mu +\alpha &{}0 \\ 0&{}-\alpha &{}\mu +\eta (\upsilon )\\ \end{array} \right] . \end{aligned}$$

By inserting the DFE point (*N*, 0, 0) into $FV^{-1}$, we obtain the following result, $R_0=\frac{\beta (\upsilon )\alpha N}{(\mu +\beta (\upsilon ))(\mu +\alpha )}$.

The analysis of $R_0$, which is dependent on the virus load, can be conducted as follows:^52^

**Case 1.** If $\upsilon <\upsilon _m$, then from Eq. (12) we get $\beta (\upsilon )=0$ and we have $R_0=0$. This corresponds to the scenario where the population is free of the measles virus, resulting in the complete eradication of the disease.

**Case 2.** If $\upsilon _m\le \upsilon \le \upsilon _0$, then from Eq. (12) we get $\beta (\upsilon )=\frac{\upsilon -\upsilon _m}{\upsilon _0-\upsilon _m}$ and we have $R_0=\frac{\beta (\upsilon )\alpha N}{(\mu +\beta (\upsilon ))(\mu +\alpha )}$.

**Case 3.** If $\upsilon _0<\nu <\upsilon _M$, then from Eq. (12) we get $\beta (\upsilon )=1$ and we have $R_0=\frac{\alpha N}{(\mu +1)(\mu +\alpha )}$.

Cases 2 and 3 are instances where the virus is present in the community and spreads widely. $R_0$ can be represented as a triangular fuzzy number (TFN) in the following manner:

$$\begin{aligned} R_0 (\upsilon )= & {} \left( 0, \frac{\beta (\upsilon )\alpha N}{(\mu +\beta (\upsilon ))(\mu +\alpha )}, \frac{\alpha N}{(\mu +1)(\mu +\alpha )}\right) \\ R_0^f= & {} E[R_0 (\upsilon )],\\= & {} \frac{\alpha N(2\beta (\upsilon )+1)}{4(\mu +\beta (\upsilon ))(\mu +\alpha )(\mu +1)}. \end{aligned}$$

### Sensitivity of $R_0$

The sensitivity index of a parameter is defined as^53^

$$\begin{aligned} \xi (\zeta )=\frac{\zeta }{R_0}\frac{\partial }{\partial \zeta } (R_0), \end{aligned}$$

where $\xi$ denote sensitivity of the parameter $\zeta$.

We calculate the sensitivity of *N* as

$$\xi N=\frac{N}{R_0}\frac{\partial }{\partial N} (R_0)=1$$.

Similarly, $$\xi \beta (\upsilon )=\frac{\beta (\upsilon )}{R_0}\frac{\partial }{\partial \beta (\upsilon )} (R_0)=\frac{\mu }{\mu +\beta (\upsilon )}$$.

$$\xi \alpha =\frac{\alpha }{R_0}\frac{\partial }{\partial \alpha } (R_0)=\frac{\mu }{(\mu +\beta (\upsilon ))^2}$$.

$$\xi \mu =\frac{\mu }{R_0}\frac{\partial }{\partial \mu } (R_0)=\frac{-\mu (2\mu +\beta (\upsilon )+\alpha )}{(\mu +\beta (\upsilon ))(\mu +\alpha )}$$.

These findings distinctly demonstrate that the parameter $R_0$ exhibits the sensitivity to the parameter *N*, $\beta (\upsilon )$ and $\alpha$ and it is insensitive to the parameter $\mu$.

### Equilibrium analysis of the model

The model mentioned above has two disease-existing endemic equilibrium points as well as a disease-free equilibrium point (DFE).

**Case 1.** If $\upsilon <\upsilon _m$, then from Eq. (12) we get $\beta (\upsilon )=0$ and

$$\begin{aligned} C^0=( S^0,E^0,I^0)=(N,0,0) \end{aligned}$$

can be obtained, which is the DFE point.

**Case 2.** If $\upsilon _m\le \upsilon \le \upsilon _0$, then $\beta (\upsilon )=\frac{\upsilon -\upsilon _m}{\upsilon _0-\upsilon _m}$ and $C^*=( S^*,E^*,I^*)$, where $S^*=\frac{N}{R_0}$, $E^*=\frac{\mu N}{\mu +\alpha } \big (1-\frac{1}{R_0} \big )$ and $I^*=\frac{\mu }{\beta (\upsilon )}$, can be obtained, where $R_0=\frac{\beta (\upsilon )\alpha N}{(\mu +\beta (\upsilon ))(\mu +\alpha )}$.

**Case 3.** If $$\upsilon _0<\nu <\upsilon _M$$, then $$\beta (\upsilon )=1$$ and $$C^{**}=( S^{**},E^{**},I^{**})$$, where $$S^{**}=\frac{N}{R_0}$$, $$E^{**}=\frac{\mu N}{\mu +\alpha } \big (1-\frac{1}{R_0} \big )$$ and $$I^{**}=\mu$$, is obtained, where $$R_0=\frac{\beta (\upsilon )\alpha N}{(\mu +\beta (\upsilon ))(\mu +\alpha )}$$.

## Numerical modeling of fuzzy SEIR model

### Forward Euler’s scheme

The forward Euler method is a numerical approximation approach for numerically solving ordinary differential equations (ODEs)^54^. It is a simple and extensively used approach for approximating the solution by stepping ahead in tiny increments iteratively. The forward Euler scheme for the studied model can be written as

14 $$\begin{aligned} {s^{n+1}}= & {} s^n+h[\mu N-s^n(\beta (\upsilon ) i^n+\mu )], \end{aligned}$$

15 $$\begin{aligned} {e^{n+1}}= & {} {e^n+h[\beta (\upsilon ) s^ni^n-(\mu +\alpha ) e^n]}, \end{aligned}$$

16 $$\begin{aligned} {i^{n+1}}= & {} {i^n+h{[\alpha e^n-(\mu +\eta (\upsilon )) i^n]}}. \end{aligned}$$

**Case 1.** If $\upsilon <\upsilon _m$, then $\beta (\upsilon )=0$ and we have

17 $$\begin{aligned} {s^{n+1}}= & {} s^n+h(\mu N- \mu s^n), \end{aligned}$$

18 $$\begin{aligned} {e^{n+1}}= & {} {e^n-h(\mu +\alpha ) e^n}, \end{aligned}$$

19 $$\begin{aligned} {i^{n+1}}= & {} {i^n+h{[\alpha e^n-(\mu +\eta (\upsilon )) i^n]}}. \end{aligned}$$

**Case 2.** If $\upsilon _m\le \upsilon \le \upsilon _0$, then $\beta (\upsilon )=\frac{\upsilon -\upsilon _m}{\upsilon _0-\upsilon _m}$ and and we have

20 $$\begin{aligned} {s^{n+1}}= & {} s^n+h[\mu N-s^n(\beta (\upsilon ) i^n+\mu )], \end{aligned}$$

21 $$\begin{aligned} {e^{n+1}}= & {} {e^n+h[\beta (\upsilon ) s^ni^n-(\mu +\alpha ) e^n]}, \end{aligned}$$

22 $$\begin{aligned} {i^{n+1}}= & {} {i^n+h{[\alpha e^n-(\mu +\eta (\upsilon )) i^n]}}. \end{aligned}$$

**Case 3.** If $\upsilon _0<\nu <\upsilon _M$, then $\beta (\upsilon )=1$ and and we have

23 $$\begin{aligned} {s^{n+1}}= & {} s^n+h[\mu N-s^n(i^n+\mu )], \end{aligned}$$

24 $$\begin{aligned} {e^{n+1}}= & {} {e^n+h[ s^ni^n-(\mu +\alpha ) e^n]}, \end{aligned}$$

25 $$\begin{aligned} {i^{n+1}}= & {} {i^n+h{[\alpha e^n-(\mu +\eta (\upsilon )) i^n]}}. \end{aligned}$$

### RK-4 method

The RK-4 method is a popular approach for solving ordinary differential equations. It has a better level of precision than the Euler method. We construct an explicit RK-4 technique for the studied model as follows:

**Step 1:**

26 $$\begin{aligned} {k_1}= & {} {h[\mu N-s^n(\beta (\upsilon ) i^n-\mu )]} \end{aligned}$$

27 $$\begin{aligned} m_1= & {} h[\beta (\upsilon ) s^ni^n-(\mu +\alpha ) e^n] \end{aligned}$$

28 $$\begin{aligned} {n_1}= & {} {h{[\alpha e^n-(\mu +\eta (\upsilon )) i^n]}} \end{aligned}$$

**Step 2:**

29 $$\begin{aligned} {k_2}= & {} {h \bigg [\mu N-\bigg (s^n+\frac{k_1}{2}\bigg )\bigg \{\beta (\upsilon ) \bigg (i^n+\frac{n_1}{2}\bigg )-\mu \bigg \}\bigg ]} \end{aligned}$$

30 $$\begin{aligned} m_2= & {} h\bigg [\beta (\upsilon ) \bigg (s^n+\frac{k_1}{2}\bigg )\bigg (i^n+\frac{n_1}{2}\bigg )-(\mu +\alpha ) \bigg (e^n+\frac{m_1}{2}\bigg )\bigg ] \end{aligned}$$

31 $$\begin{aligned} {n_2}= & {} {h{\bigg [\alpha \bigg (e^n+\frac{m_1}{2}\bigg )-(\mu +\eta (\upsilon )) \bigg (i^n+\frac{n_1}{2}\bigg )\bigg ]}} \end{aligned}$$

**Step 3:**

32 $$\begin{aligned} {k_3}= & {} {h\bigg [\mu N-\bigg (s^n+\frac{k_2}{2}\bigg )\bigg \{\beta (\upsilon ) \bigg (i^n+\frac{n_2}{2}\bigg )-\mu \bigg \}\bigg ]} \end{aligned}$$

33 $$\begin{aligned} m_3= & {} h\bigg [\beta (\upsilon ) \bigg (s^n+\frac{k_2}{2}\bigg )\bigg (i^n+\frac{n_2}{2}\bigg )-(\mu +\alpha ) \bigg (e^n+\frac{m_2}{2}\bigg )\bigg ] \end{aligned}$$

34 $$\begin{aligned} {n_3}= & {} {h{\bigg [\alpha \bigg (e^n+\frac{m_2}{2}\bigg )-(\mu +\eta (\upsilon )) \bigg (i^n+\frac{n_2}{2}\bigg )\bigg ]}} \end{aligned}$$

**Step 4:**

35 $$\begin{aligned} {k_4}= & {} {h[\mu N-(s^n+k_3)\{\beta (\upsilon ) (i^n+n_3)-\mu \}]} \end{aligned}$$

36 $$\begin{aligned} m_4= & {} h[\beta (\upsilon ) (s^n+k_3)(i^n+n_3)-(\mu +\alpha ) (e^n+m_3)] \end{aligned}$$

37 $$\begin{aligned} {n_4}= & {} {h{[\alpha (e^n+m_3)-(\mu +\eta (\upsilon )) (i^n+n_3)]}} \end{aligned}$$

**Final step:**

38 $$\begin{aligned} {s^{n+1}}= & {} {s^n+\frac{1}{6}{[k_1+2{k_2}+2{k_3}+{k_4}]}} \end{aligned}$$

39 $$\begin{aligned} {e^{n+1}}= & {} {e^n+\frac{1}{6}{[m_1+2{m_2}+2{m_3}+{m_4}]}} \end{aligned}$$

40 $$\begin{aligned} {i^{n+1}}= & {} {i^n+\frac{1}{6}{[n_1+2{n_2}+2{n_3}+{n_4}]}} \end{aligned}$$

### NSFD scheme

The NSFD scheme is a comprehensive collection of numerical methods utilized to obtain numerical solutions for differential equations through the creation of a discrete model. Initially introduced by Mickens^3^, the NSFD scheme aimed to enhance the precision and stability of numerical solutions. The specific formulation of the NSFD scheme for the given model can be expressed as follows

41 $$\begin{aligned} {s^{n+1}}= & {} {\frac{s^n+h\mu N}{1+h(\beta (\upsilon )i^n+\mu )}}, \end{aligned}$$

42 $$\begin{aligned} {e^{n+1}}= & {} {\frac{e^n+h\beta (\upsilon ) s^{n}i^{n}}{1+h(\mu +\alpha )}}, \end{aligned}$$

43 $$\begin{aligned} {i^{n+1}}= & {} {\frac{i^n+h\alpha e^n}{1+h(\mu +\eta (\upsilon ))}}. \end{aligned}$$

**Case 1.** If $\upsilon <\upsilon _m$, then $\beta (\upsilon )=0$ and the system (41–43) becomes

44 $$\begin{aligned} {s^{n+1}}= & {} {\frac{s^n+h\mu N}{1+h\mu }}, \end{aligned}$$

45 $$\begin{aligned} {e^{n+1}}= & {} {\frac{e^n}{1+h(\mu +\alpha )}}, \end{aligned}$$

46 $$\begin{aligned} {i^{n+1}}= & {} {\frac{i^n+h\alpha e^n}{1+h(\mu +\eta (\upsilon ))}}. \end{aligned}$$

**Case 2.** If $\upsilon _m\le \upsilon \le \upsilon _0$, then $\beta (\upsilon )=\frac{\upsilon -\upsilon _m}{\upsilon _0-\upsilon _m}$ and the system (41–43) becomes

47 $$\begin{aligned} {s^{n+1}}= & {} {\frac{s^n+h\mu N}{1+h(\beta (\upsilon )i^n+\mu )}}, \end{aligned}$$

48 $$\begin{aligned} {e^{n+1}}= & {} {\frac{e^n+h\beta (\upsilon ) s^{n}i^{n}}{1+h(\mu +\alpha )}}, \end{aligned}$$

49 $$\begin{aligned} {i^{n+1}}= & {} {\frac{i^n+h\alpha e^n}{1+h(\mu +\eta (\upsilon ))}}. \end{aligned}$$

**Case 3.** If $\upsilon _0<\nu <\upsilon _M$, then $\beta (\upsilon )=1$ and the system (41–43) becomes

50 $$\begin{aligned} {s^{n+1}}= & {} {\frac{s^n+h\mu N}{1+h(i^n+\mu )}}, \end{aligned}$$

51 $$\begin{aligned} {e^{n+1}}= & {} {\frac{e^n+h s^{n}i^{n}}{1+h(\mu +\alpha )}}, \end{aligned}$$

52 $$\begin{aligned} {i^{n+1}}= & {} {\frac{i^n+h\alpha e^n}{1+h(\mu +\eta (\upsilon ))}}. \end{aligned}$$

### Convergence analysis of the NSFD scheme

Let

53 $$\begin{aligned} F= & {} {\frac{s+h\mu N}{1+h(\beta (\upsilon )i+\mu )}}, \end{aligned}$$

54 $$\begin{aligned} G= & {} {\frac{e+h\beta (\upsilon ) si}{1+h(\mu +\alpha )}}, \end{aligned}$$

and

55 $$\begin{aligned} H={\frac{i+h\alpha e}{1+h(\mu +\eta (\upsilon ))}}. \end{aligned}$$

Jacobean matrix of Eqs. (53–55) is,

56 $$\begin{aligned} J= & {} \left[ \begin{array}{cccc} F_s &{} F_e &{} F_i \\ G_s &{} G_e &{} G_i \\ H_s &{} H_e&{} H_i \\ \end{array} \right] \end{aligned}$$

57 $$\begin{aligned} J= & {} \left[ \begin{array}{cccc} \frac{1}{1 + h (\mu +\beta (\upsilon )) }&{} 0 &{} 0 \\ \frac{h\beta (\upsilon )i}{1 + h (\mu +\alpha ) } &{} \frac{1}{1 + h (\mu +\alpha ) } &{} \frac{h\beta (\upsilon )s}{1 + h (\mu +\alpha ) } \\ 0 &{} \frac{h\alpha }{1 + h (\mu +\eta (\upsilon )) } &{} \frac{1}{1 + h (\mu +\eta (\upsilon )) }\\ \end{array} \right] \end{aligned}$$

Jacobean at the DFE point (*N*, 0, 0) is

58 $$\begin{aligned} J_1=\left[ \begin{array}{cccc} \frac{1}{1 + h \mu }&{} 0 &{} 0 \\ 0 &{} \frac{1}{1 + h (\mu +\alpha ) } &{} 0 \\ 0 &{} \frac{h\alpha }{1 + h (\mu +\eta (\upsilon )) } &{} \frac{1}{1 + h (\mu +\eta (\upsilon )) }\\ \end{array} \right] \end{aligned}$$

Here $\lambda _1=\frac{1}{1 +h\mu }<1$, $\lambda _2=\frac{1}{1 + h (\mu +\alpha ) }<1$ and $\lambda _3=\frac{1}{1 + h (\mu +\eta (\upsilon )) }<1$ which shows that the NSFD method unconditionally converges.

### Consistency analysis

Consistency plays a crucial role in numerical schemes as it establishes a connection between the discrete equations and the continuous system they represent. Taylor’s series is utilized to discretize derivative operators in differential equations, with higher-order terms intentionally eliminated to achieve the desired accuracy. The excluded terms give rise to an error known as the truncation error or discretization error in the solution of the given system. Consistency is a fundamental characteristic that guarantees the diminishing of the discretization error towards zero as the mesh size and time steps approach zero. Taylor series for the susceptible compartment can be written as

59 $$\begin{aligned} s^{n+1}=s^n+h\frac{ds}{dt}+\frac{h^2}{2!}\frac{d^2s}{dt^2}+\frac{h^3}{3!}\frac{d^3s}{dt^3}+... \end{aligned}$$

From Eq. (41), we have

60 $$\begin{aligned} s^{n+1}\left( 1+h(\beta (\upsilon )i^n+\mu )\right) =s^n+h\mu N. \end{aligned}$$

$$\begin{aligned} \left( s^n+h\frac{ds}{dt}+\frac{h^2}{2!}\frac{d^2s}{dt^2}+\frac{h^3}{3!}\frac{d^3s}{dt^3}+... \right) \Big (1+h(\beta (\upsilon )i^n+\mu ) \Big )=s^n+h\mu N, \end{aligned}$$.

$$\begin{aligned} s^n+h\beta (\upsilon )i^ns^n+h\mu s^n+h\frac{ds}{dt}+h^2\frac{ds}{dt}=s^n+h\mu N. \end{aligned}$$

Taking $h\rightarrow 0$, we get

$$\begin{aligned} {\frac{dS}{dt}}={\mu N-\beta (\upsilon ) SI-\mu S}. \end{aligned}$$

Similarly, Taylor series for the exposed compartment can be written as ‘

61 $$\begin{aligned} e^{n+1}=e^n+h\frac{de}{dt}+\frac{h^2}{2!}\frac{d^2e}{dt^2}+\frac{h^3}{3!}\frac{d^3e}{dt^3}+ \ldots \end{aligned}$$

Equation (42) can be written as

62 $$\begin{aligned} e^{n+1}\left( 1+h\mu +h\alpha ) \right) =e^n+h\beta (\upsilon ) s^{n}i^{n}. \end{aligned}$$

$$\begin{aligned} \left( e^n+h\frac{de}{dt}+\frac{h^2}{2!}\frac{d^2e}{dt^2}+\frac{h^3}{3!}\frac{d^3e}{dt^3}+.. \right) \Big (1+h\mu +h\alpha )\Big )=e^n+h\beta (\upsilon ) s^{n}i^{n}\\ e^n+e^nh\mu +e^nh\alpha +h\frac{de}{dt}+h^2 \mu \frac{de}{dt}+h^2 \alpha \frac{de}{dt}+ \ldots =e^n+h\beta (\upsilon ) s^{n}i^{n}. \end{aligned}$$

Taking $h\rightarrow 0$, we get

$$\begin{aligned} \frac{dE}{dt}={\beta (\upsilon ) SI-(\mu +\alpha ) E}. \end{aligned}$$

Similarly, applying the Taylor series Eq. (43) gives

$$\begin{aligned} \frac{dI}{dt}={\alpha E-(\mu +\eta (\upsilon )) I}. \end{aligned}$$

The above results show that the proposed method is first-order accurate. Using the Taylor expansion, the forward Euler scheme is likewise consistent with order 1. However, some numerical methods, such as the unconditionally positive finite difference (UPFD) scheme, have a flaw in terms of consistency. To ensure the consistency of the UPFD scheme, the time step that depends on the spatial step must be designed in such a way that the truncation error is reduced to zero^55^.

## Numerical simulations

Table 1 displays the values of parameters used for numerical simulations while the initial conditions are given in Table 2.

**Table 1:** Values of parameters.

Parameters	Values
*N*	0.5
$\mu$	0.02
$\beta (\nu )$	Fuzzy number (adjusted)
$\eta (\nu )$	Fuzzy number (adjusted)
$\alpha$	0.4

**Table 2:** Values of initial conditions.

Symbols	Values
*S*(0)	0.2
*E*(0)	0.3
*I*(0)	0.5
*R*(0)	0.1

**Figure 2:**
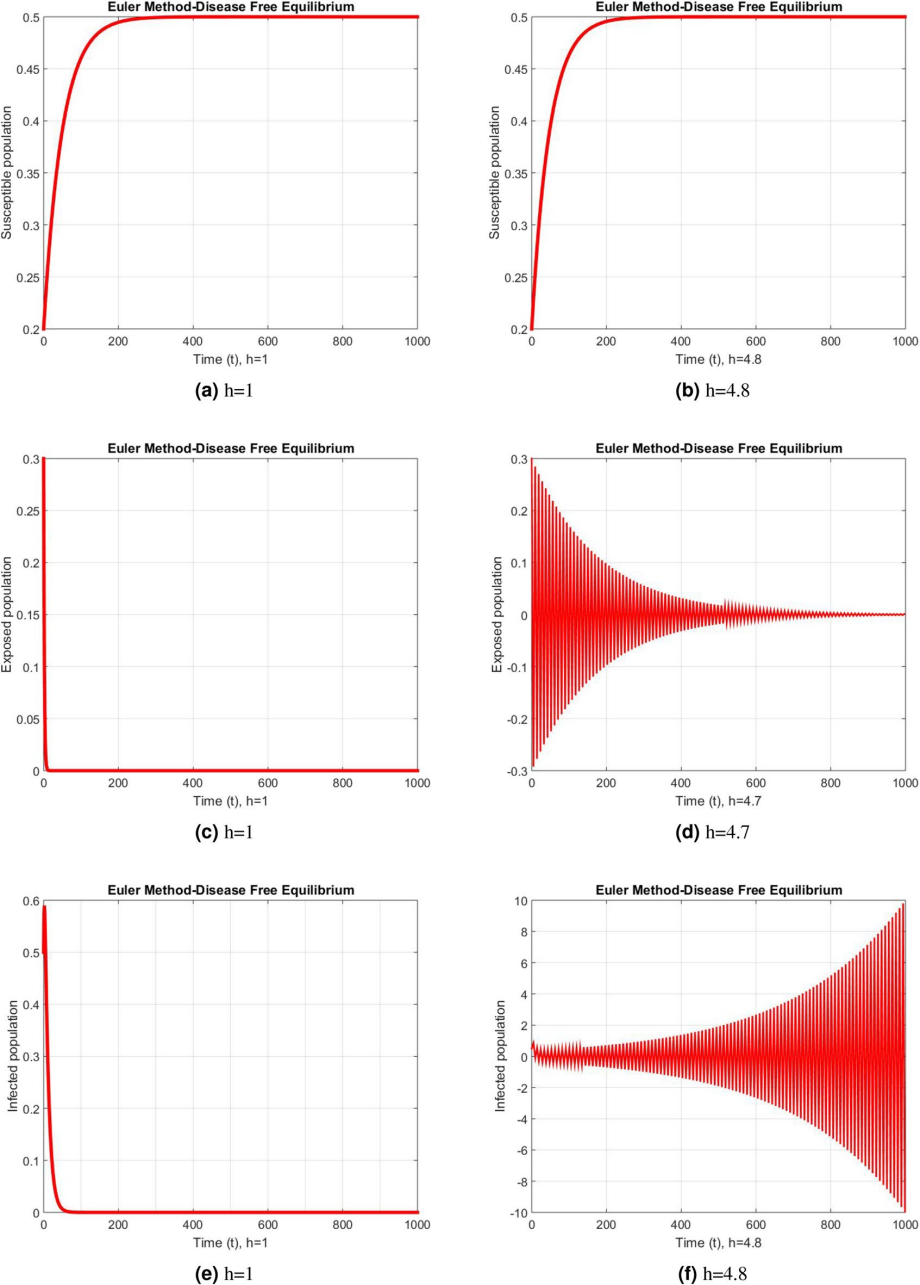
Subpopulation for case 1 at different step sizes using Euler method.

**Figure 3:**
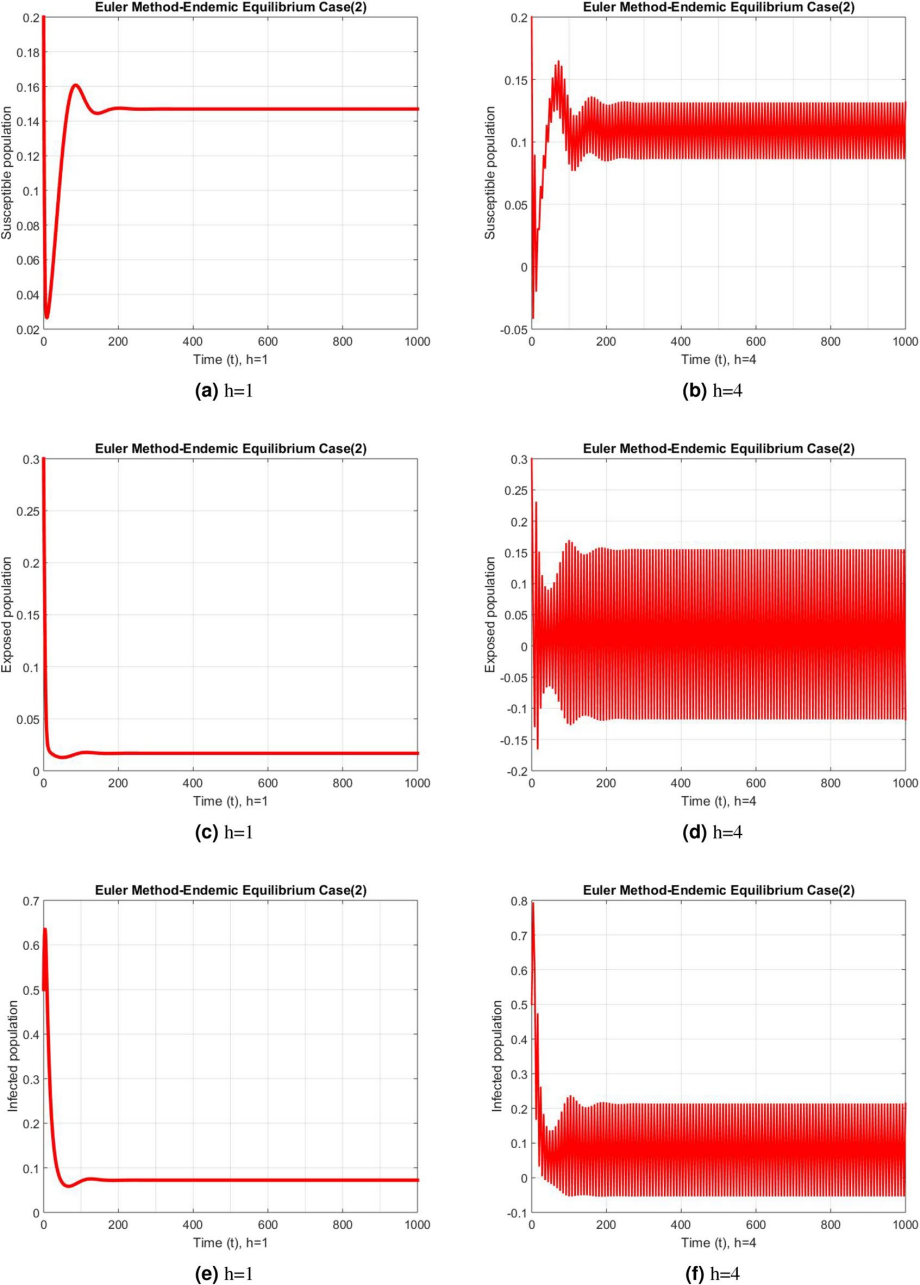
Subpopulation for case 2 at different step sizes using Euler method.

**Figure 4:**
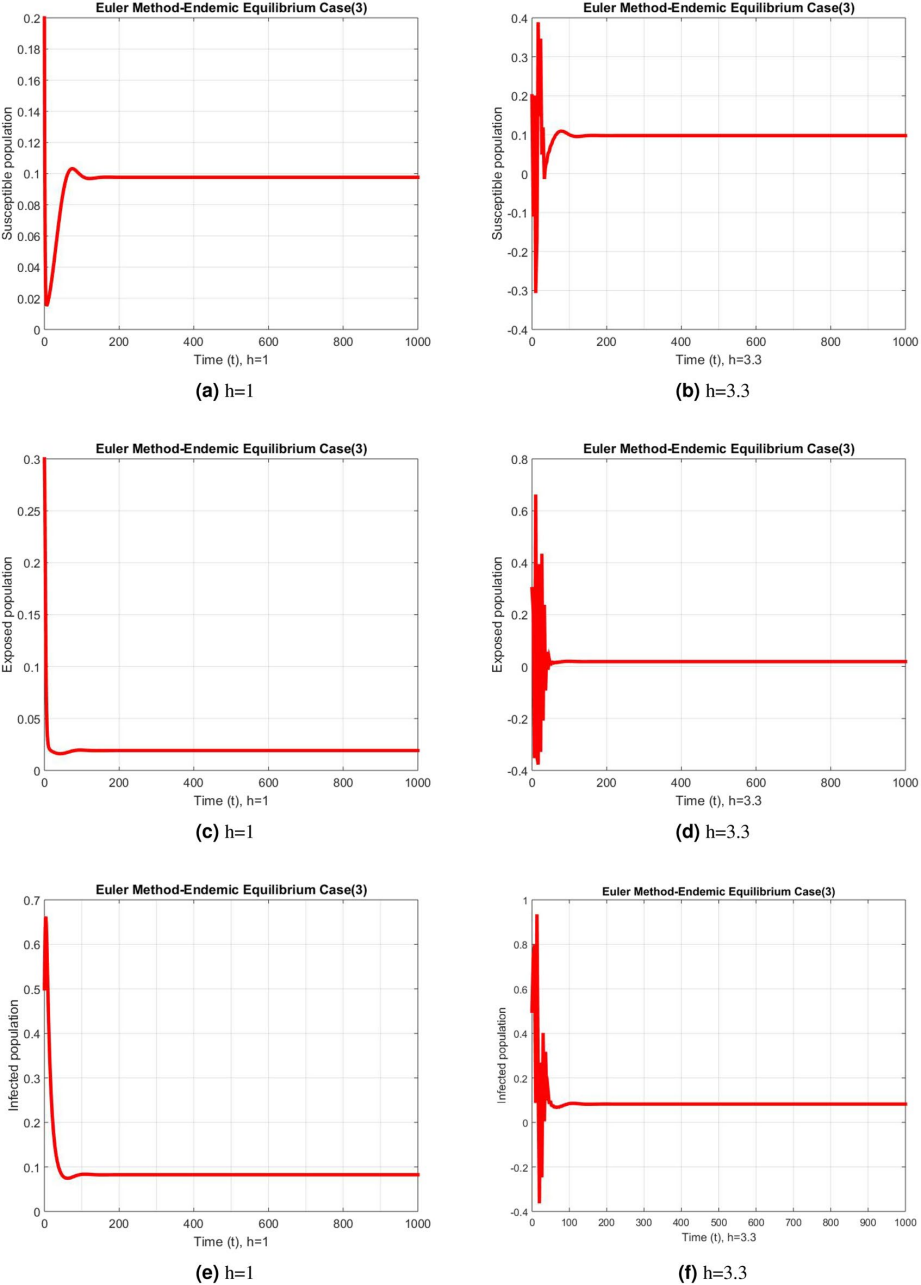
Subpopulation for case 3 at different step sizes using Euler method.

**Figure 5:**
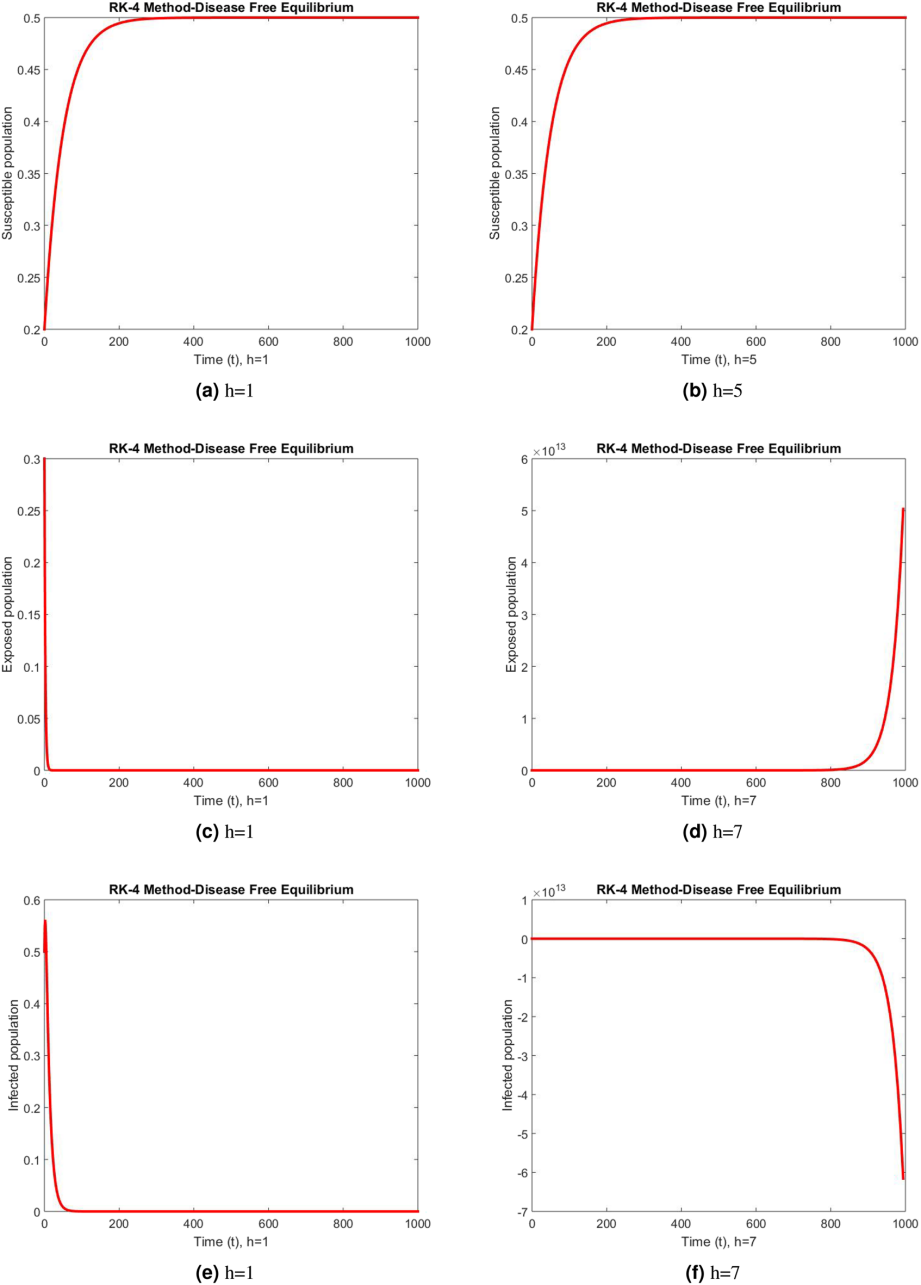
Subpopulation at DFE using RK-4 method.

**Figure 6:**
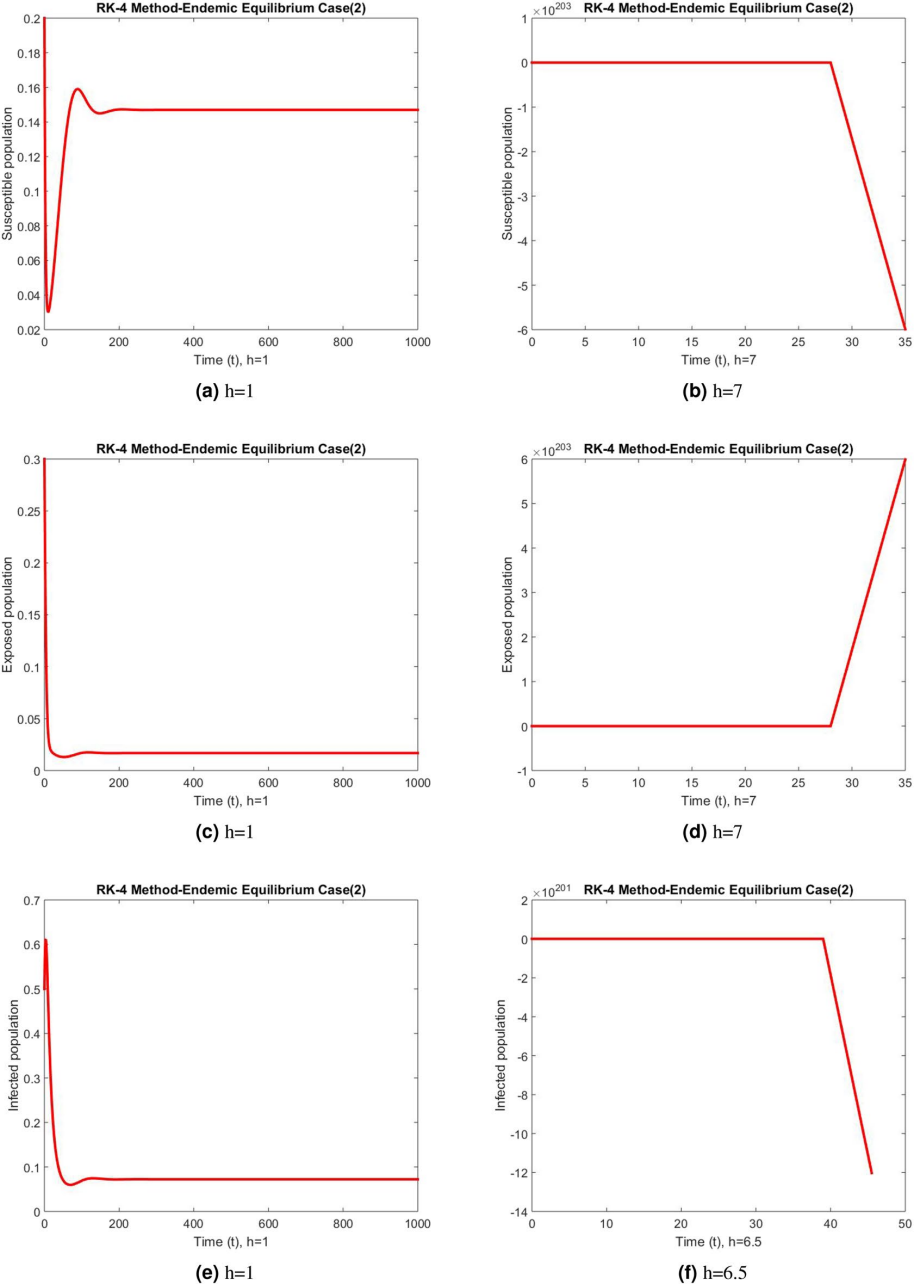
Subpopulation for case 2 at different step sizes using RK-4 method.

**Figure 7:**
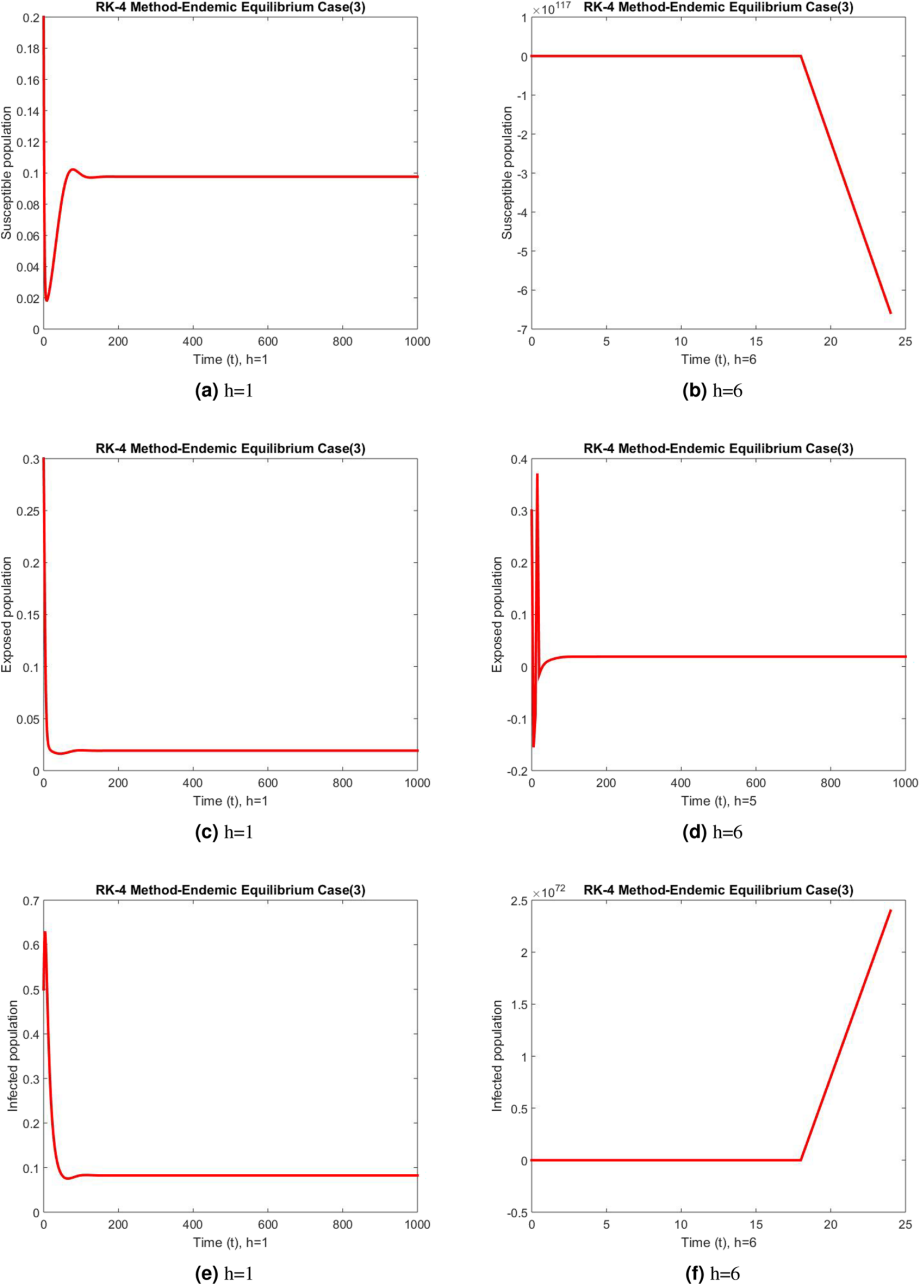
Subpopulation for case 3 at different step sizes using RK-4 method.

**Figure 8:**
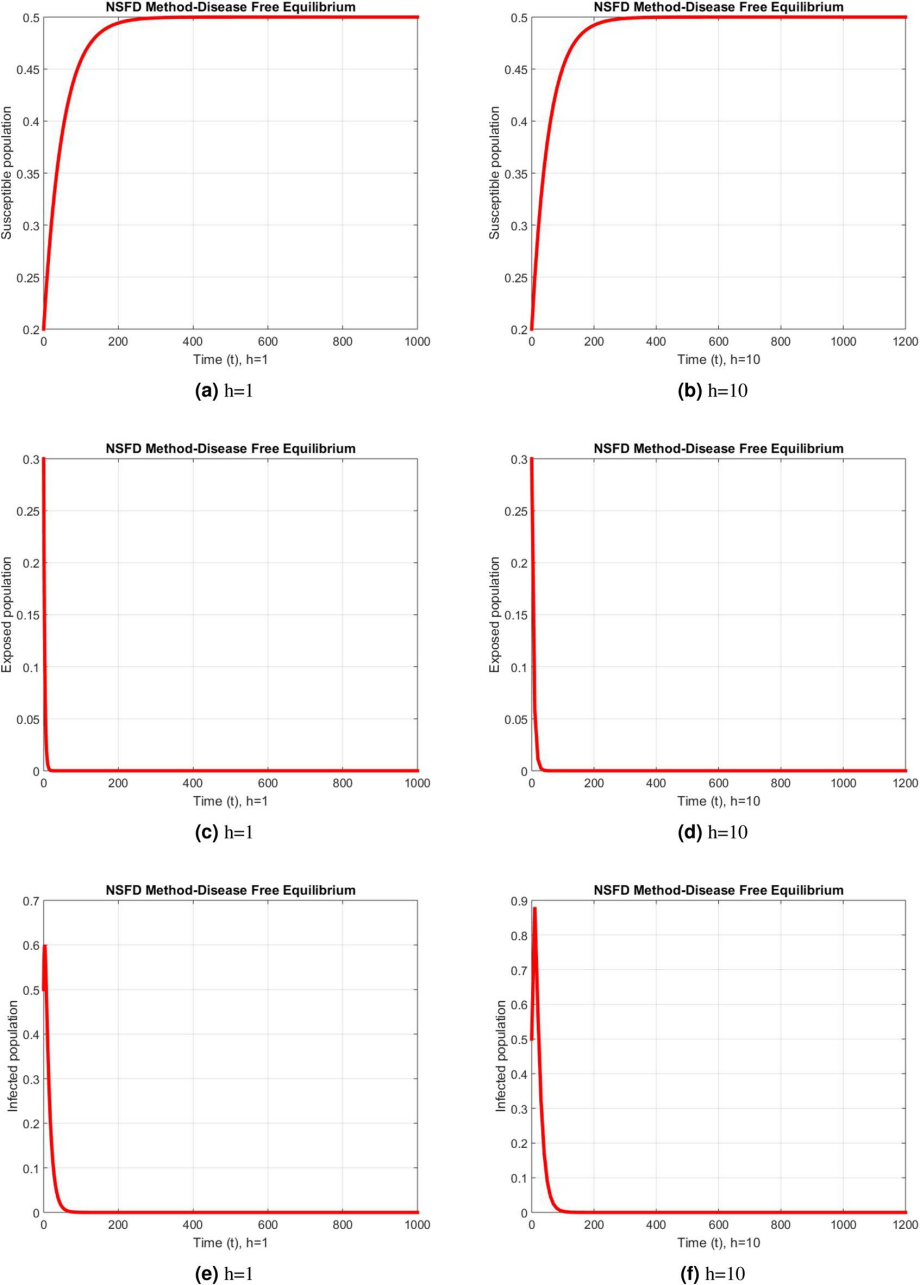
Subpopulation for case 1 at different step sizes using NSFD method.

**Figure 9:**
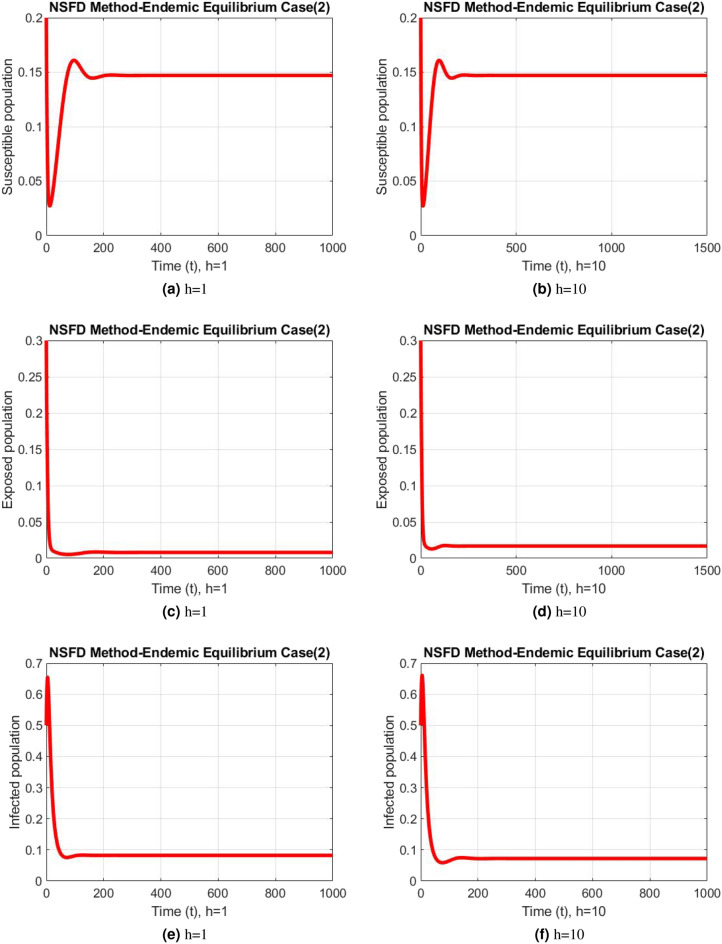
Subpopulation for case 2 at different step sizes using NSFD method.

**Figure 10:**
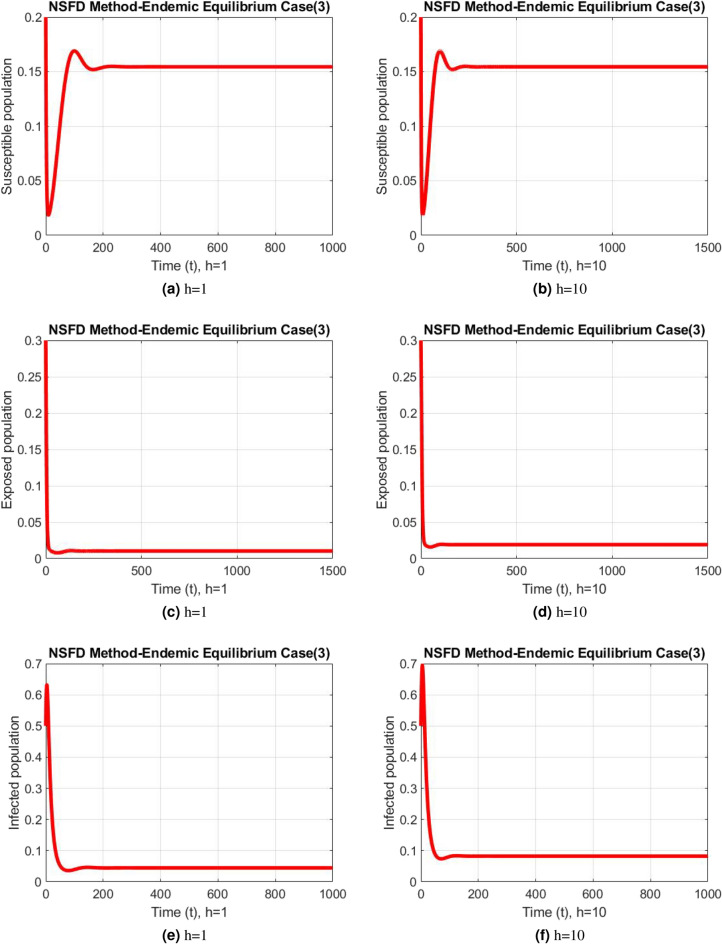
Subpopulation for case 3 at different step sizes using NSFD method.

The graphs above depict the behavior of the fuzzy SEIR model. At a small step size of h = 1, Fig. 2 demonstrates the favorable behavior and convergence of the Euler method at the DFE point. The method starts producing non physical oscillations and negative values as the step increases. Negative values in disease dynamical models are meaningless as these compartments consist of populations that cannot be negative. As a result of this behavior, it is possible to conclude that the approach is incapable of representing disease dynamics in a fuzzy context. The dynamics of subpopulations for case 2 using the Euler method are shown in Fig. 3 at different step sizes. The method initially remains positive, shows stable behavior and converges to the true equilibrium points at small step sizes. As soon as the value of the step size is increased, the methods started oscillating non physically and negative values are also produced. This behavior shows the drawback of the method and we concluded that the method is not a good tool to represent the disease dynamical models. Figure 4 shows the graphical behavior of the subpopulations for case 3 using the Euler method. The results are not different from the first two cases as the method exhibits stable and converging behavior at the start at smaller values of the step size. An increase in the values of the step sizes produces negative values. Since any of these subpopulations cannot be negative, the method fails to show the dynamics of the model in this case too. Figure 5 depicts the behavior of subpopulations using the RK-4 technique at various step sizes, with an emphasis on the DFE point. When the step size is reduced, it is clear that the solutions of the investigated model slowly approach their equilibrium states for all three situations. A small rise in step size, on the other hand, causes the algorithm to deviate from this convergence pattern in all cases. The graphical results of the RK-4 technique for cases 2 and 3 are shown in Figs. 6 and 7, respectively. When using smaller step sizes, the technique retains its positive nature and displays convergence in both cases. However, as the step size is increased, the approach begins to generate negative values and exhibit divergence. The maintenance of positivity is critical inside epidemic models since negative values have no meaningful interpretation in this environment. As the step size is raised, the RK-4 approach fails to maintain this positive assumption. As a result, changes in the time step sizes have a direct impact on the scheme’s convergence. This event emphasizes the method’s limitations in analyzing the model’s long-term dynamics. Based on this behavior, it is clear that the RK-4 approach is unsuitable for studying disease epidemic dynamics. The numerical results of the NSFD scheme at the DFE point are presented in Fig. 8. The method remains positive, stable and convergent at both smaller and larger step sizes. Interestingly, the increase in the step size does not change the positivity and convergence of the method. We concluded that the method is independent of the values of the step size. This is an important feature that classical methods do not preserve. The numerical simulations for case 2 and case 3 of the NSFD method are presented in Figs. 9 and 10 respectively. In both cases, all graphs are converging to the same equilibrium points at both smaller and larger values of the step sizes. The increase in the values of the step sizes does not create any change in the convergence and positivity of the NSFD method. This behavior of the scheme makes it superior over the other two schemes. It is concluded that the proposed method is capable to describe the disease dynamics in fuzzy conditions. It is clear from the graphs that all of the schemes show similar behaviors at small step sizes and converge to the same equilibrium points. The Euler method and RK-4 techniques show oscillations and negative behavior as we increase the step size while the NSFD method still converges to the same point. The graphical behaviors demonstrate that the Euler and RK-4 techniques only provide a convergence solution for small step sizes and are unable to converge for large step sizes. The NSFD scheme, on the other hand, exhibits good behavior and provides a convergent solution for very large values of step sizes.

## Conclusion

Measles is a highly contagious viral disease caused by the Measles virus (MeV). It mostly affects the respiratory system, although it can cause serious consequences, particularly in young children and immunocompromised people. Mathematical modeling is a valuable tool for understanding measles transmission dynamics, predicting outbreak patterns, and guiding effective control tactics. It assists researchers and policymakers in understanding how measles spreads, estimating potential consequences, and evaluating intervention efforts. Measles is studied using a variety of mathematical models. A fuzzy SEIR model of measles is discussed in this article. The rates of disease transmission and recovery are treated as fuzzy sets. In our assumptions, we considered that the transmission of the disease by infected individuals is not uniform, and each person exhibits a distinct level of infectivity based on their virus quantity. Likewise, the recovery rate varies among individuals and is not uniform. As fuzzy variables are influenced by the virus load, which is contingent upon the virus quantities, we conducted an analysis for various virus amounts. In light of this, we examined the fuzzy equilibrium points of the model under consideration, taking into account the virus quantities within the population. Our findings demonstrated that when the virus amount falls below the minimum threshold required for disease transmission, the DFE point is attained. Conversely, when the virus amounts surpass the minimum threshold, we observed the emergence of endemic equilibrium points. By employing the next-generation matrix method, we computed the basic reproduction number, and its sensitivity is discussed. We further derived the fuzzy basic reproduction number by utilizing the expected value of a fuzzy number. The fuzzy SEIR model is then numerically solved using three distinct approaches, namely the forward Euler, the RK-4 and the NSFD methods. Following a comparison of the three methods’ results, it becomes clear that the proposed NSFD technique accurately captures the convergence problem for each time step size. In contrast, the classical Euler and the RK-4 methods exhibit an unlimited solution even for very tiny time steps. The NSFD technique exhibits stable behavior numerically and exhibits a good agreement with analytic results held by the continuous model since it is an explicit numerical scheme and is thus simple to apply. The suggested approach is applicable for big step sizes and maintains all significant characteristics that demonstrate the method’s effectiveness. To support our theoretical findings, we have carried out a few numerical tests. The findings indicate that, in comparison to the similar crisp model, the fuzzy SEIR model is more realistic. The developed scheme underwent analysis for varying virus quantities. It was essential to preserve solution positivity in dynamic population models, which was effectively addressed by the proposed numerical technique, as demonstrated in this article. The results highlighted that the proposed method successfully maintained solution positivity across different virus quantities, irrespective of whether small or large step sizes were employed. The essential characteristics of epidemic models include convergence and consistency. The proposed NSFD method successfully maintains these features, ensuring the model’s convergence towards accurate solutions and maintaining consistency with the underlying continuous dynamical system. Saturated incidence, treatment, delayed, fractional, and stochastic directions can all be considered in future work. This research can be expanded to include other aspects such as age structure and interventions such as vaccine campaigns or control measures.

## Data Availability

The datasets analyzed during the current study are available from the corresponding author upon reasonable request.
